# Cumulative Viral Load and Virologic Decay Patterns after Antiretroviral Therapy in HIV-Infected Subjects Influence CD4 Recovery and AIDS

**DOI:** 10.1371/journal.pone.0017956

**Published:** 2011-05-20

**Authors:** Vincent C. Marconi, Greg Grandits, Jason F. Okulicz, Glenn Wortmann, Anuradha Ganesan, Nancy Crum-Cianflone, Michael Polis, Michael Landrum, Matthew J. Dolan, Sunil K. Ahuja, Brian Agan, Hemant Kulkarni, the Infectious Disease Clinical Research Program (IDCRP) HIV Working Group

**Affiliations:** 1 Division of Infectious Diseases, Emory University School of Medicine, Atlanta, Georgia, United States of America; 2 Infectious Disease Clinical Research Program, Uniformed Services University of the Health Sciences, Bethesda, Maryland, United States of America; 3 Division of Biostatistics, University of Minnesota, Minneapolis, Minnesota, United States of America; 4 Infectious Disease Service, San Antonio Military Medical Center, Brooke Army Medical Center, Fort Sam Houston, Texas, United States of America; 5 Infectious Disease Service, Walter Reed Army Medical Center, Washington, D.C., United States of America; 6 Infectious Disease Clinic, National Naval Medical Center, Bethesda, Maryland, United States of America; 7 Infectious Disease Clinic, Naval Medical Center San Diego, San Diego, California, United States of America; 8 National Institute for Allergy and Infectious Diseases, National Institutes of Health, Bethesda, Maryland, United States of America; 9 Henry M. Jackson Foundation, Wilford Hall United States Air Force Medical Center, Lackland Air Force Base, Texas, United States of America; 10 Veterans Administration Research Center for AIDS and HIV-1 Infection, South Texas Veterans Health Care System, San Antonio, Texas, United States of America; 11 Department of Medicine, University of Texas Health Science Center, San Antonio, Texas, United States of America; 12 Department of Microbiology and Immunology, and Biochemistry, University of Texas Health Science Center, San Antonio, Texas, United States of America; University of Liverpool, United Kingdom

## Abstract

**Background:**

The impact of viral load (VL) decay and cumulative VL on CD4 recovery and AIDS after highly-active antiretroviral therapy (HAART) is unknown.

**Methods and Findings:**

Three virologic kinetic parameters (first year and overall exponential VL decay constants, and first year VL slope) and cumulative VL during HAART were estimated for 2,278 patients who initiated HAART in the U.S. Military HIV Natural History Study. CD4 and VL trajectories were computed using linear and nonlinear Generalized Estimating Equations models. Multivariate Poisson and linear regression models were used to determine associations of VL parameters with CD4 recovery, adjusted for factors known to correlate with immune recovery. Cumulative VL higher than the sample median was independently associated with an increased risk of AIDS (relative risk 2.38, 95% confidence interval 1.56–3.62, p<0.001). Among patients with VL suppression, first year VL decay and slope were independent predictors of early CD4 recovery (p = 0.001) and overall gain (p<0.05). Despite VL suppression, those with slow decay during the first year of HAART as well as during the entire therapy period (overall), in general, gained less CD4 cells compared to the other subjects (133 vs. 195.4 cells/µL; p = 0.001) even after adjusting for potential confounders.

**Conclusions:**

In a cohort with free access to healthcare, independent of established predictors of AIDS and CD4 recovery during HAART, cumulative VL and virologic decay patterns were associated with AIDS and distinct aspects of CD4 reconstitution.

## Introduction

The initial goal of highly-active antiretroviral therapy (HAART) was to improve AIDS-free survival and attempt to mitigate the harmful effects of treatment. Immune reconstitution via CD4 recovery served as an intermediate marker for response to HAART because of its predictive capacity for AIDS events and death.[1], [2], [3] Thereafter, virologic suppression became the primary target for therapy because it was shown to be an appropriate, early predictor of immunologic response and clinical outcomes.[4], [5], [6], [7] Furthermore, it was demonstrated that incomplete suppression of viral replication allowed for the emergence of drug resistance and ultimately virologic failure.[8], [9] These findings led to recommendations in the U.S. Department of Health and Human Services guidelines that patients should achieve complete virologic suppression (viral load [VL] <400 copies/mL by 24 weeks or <50 copies/mL by 48 weeks) and maintain suppression thereafter.[10]

Even among patients reaching these virologic targets, there are significant inter-individual differences in the recovery of CD4+ T cells and risk of clinical events, suggesting that other factors may relate to these outcomes.[11], [12], [13], [14], [15], [16] Age at HAART initiation, pre-HAART VL and CD4 cell count, magnitude of and time to VL suppression all have been shown to influence CD4 recovery and clinical outcomes. [4], [13], [17], [18], [19], [20], [21], [22], [23], [24], [25], [26] Although the relationship of virologic decay patterns with VL changes during HAART has been described,[23], [27], [28], [29] the impact of these decay patterns on CD4 reconstitution and risk of subsequent clinical AIDS events has not been fully elucidated. Furthermore, it is also conceivable that the overall VL burden, represented as the cumulative VL during HAART, may also influence CD4 recovery and risk of AIDS events. Hence, we determined whether the patterns of virologic decay and the cumulative VL during HAART were associated with AIDS and CD4 recovery after HAART initiation independent of the currently recommended dichotomous measures of VL suppression[10] within a large, observational cohort with free access to medications and care, high rates of adherence, and low rates of injection drug use.[26], [30] If virologic decay measures are independently associated with outcomes, this could provide some explanation as to why some individuals experience inadequate treatment response despite achieving virologic suppression. Additionally, cumulative viral load could serve as a sensitive marker for risk of AIDS after HAART beyond traditional measures.

## Materials and Methods

### Study Participants

The U.S. Military HIV Natural History Study (NHS) is a prospective multicenter observational study of HIV-infected active duty military personnel and other beneficiaries (spouses, dependents, and retired military personnel) from the Army, Navy/Marines and Air Force. Seroconverters (SC) were defined as patients having a documented HIV seronegative date prior to the first positive HIV date (see Table S1). The estimated date of seroconversion for SC was defined as the midpoint between the two dates. All CD4 count, VL, and other measurements were done as part of routine clinical care.[31] The clinically-approved methodology for this testing varied by site and over time. Prior ARV use referred to any antiretroviral therapy not meeting the NHS definition of HAART.[26] HAART initiation was the date when HAART was first prescribed.

### Ethics Statement

Participants who provided written informed consent and initiated HAART through July 1, 2008 regardless of regimen continuation were included in the present study. The NHS and this substudy have been approved by each center's Institutional Review Board and the Uniformed Services University of the Health Sciences Institutional Review Board.

### Statistical Analysis

#### VL Parameters

A primary aim of this study was to capture and summarize the overall and early VL dynamics in such a manner as to permit their eventual use in clinical practice. In that regard, we made the following assumptions: i) by the time HAART is typically initiated for an individual in the NHS a natural steady state VL exists; ii) once potent HAART is initiated there is a rapid decline in the VL followed by a slower decline; and iii) such a typical pattern of VL can be explained on the basis of an exponential decay in the circulating VL. The definitions of the parameters used in this study are shown in Table S1, and the theoretical bases for the estimation of these parameters are further described in Note S1. The composite “virologic decay” refers to the application of an exponential decay equation which has been fitted to all viral loads available for an individual after the initiation of HAART. For a majority of participants in this cohort who have a high level of adherence, the virologic decay pattern corresponds to the concatenation of each "classical" (first, second, etc.) phase of decay for that individual. For some participants, their virologic decay does not follow these patterns due to suboptimal adherence, inadequate drug levels, drug resistance, and treatment interruption.

We computed four VL parameters at the level of each individual: (i–ii) exponential decay constant of VL change during entire duration of HAART (overall) and during the first year of HAART, respectively; (iii) VL slope during the first year of HAART obtained using linear Generalized Estimating Equations (GEE) models; and (iv) cumulative VL (Table S1). The VL parameters described above in i, ii, and iii are designated as VL kinetic parameters. Similarly, we computed the following four CD4 count parameters at the level of the individual: (i–ii) slope of the CD4 count change during and after the first two years of HAART; (iii) mean CD4 count after the first two years of HAART; and (iv) overall gain in CD4 counts (Table S1).

#### Cohort level analyses

The cohort-level analyses made use of all available CD4^+^ T cell count and VL data for all subjects to generate time-trend lines or curves using linear and non-linear GEE models, assuming an equal correlation structure. The time-trend curves derived by non-linear GEE modeling were refined further using spline smoothed curves with knots at the end of each year since HAART initiation. The resulting curves describe an overall or composite VL pattern for the cohort.

#### Association analyses

We estimated the parameters detailed in Table S1 for each individual. The association of these individual level parameters with the risk of AIDS (defined using 1993 clinical criteria[32] but did not include a CD4 count <200 cells/µL as an endpoint) was assessed by Poisson regression models, and with recovery of CD4 counts by linear regression models. In these models we accounted for the potential confounding due to VL suppression by HAART by including two covariates - achievement of VL suppression (as defined in Table S1) and the time taken to achieve VL suppression from the start of HAART. As described in the results, we ran these multivariate analyses for the VL parameters that were estimated (i) by including all VL measurements after HAART and separately (ii) by restricting to only those measurements after HAART but prior to the occurrence of the first AIDS event. Statistical significance was evaluated at a type I error rate of 0.05. All statistical analyses were conducted using Stata 7.0 (Stata Corp., College Station, TX).

## Results

### Cohort-level VL and CD4 changes after HAART initiation

**Figure 1:**
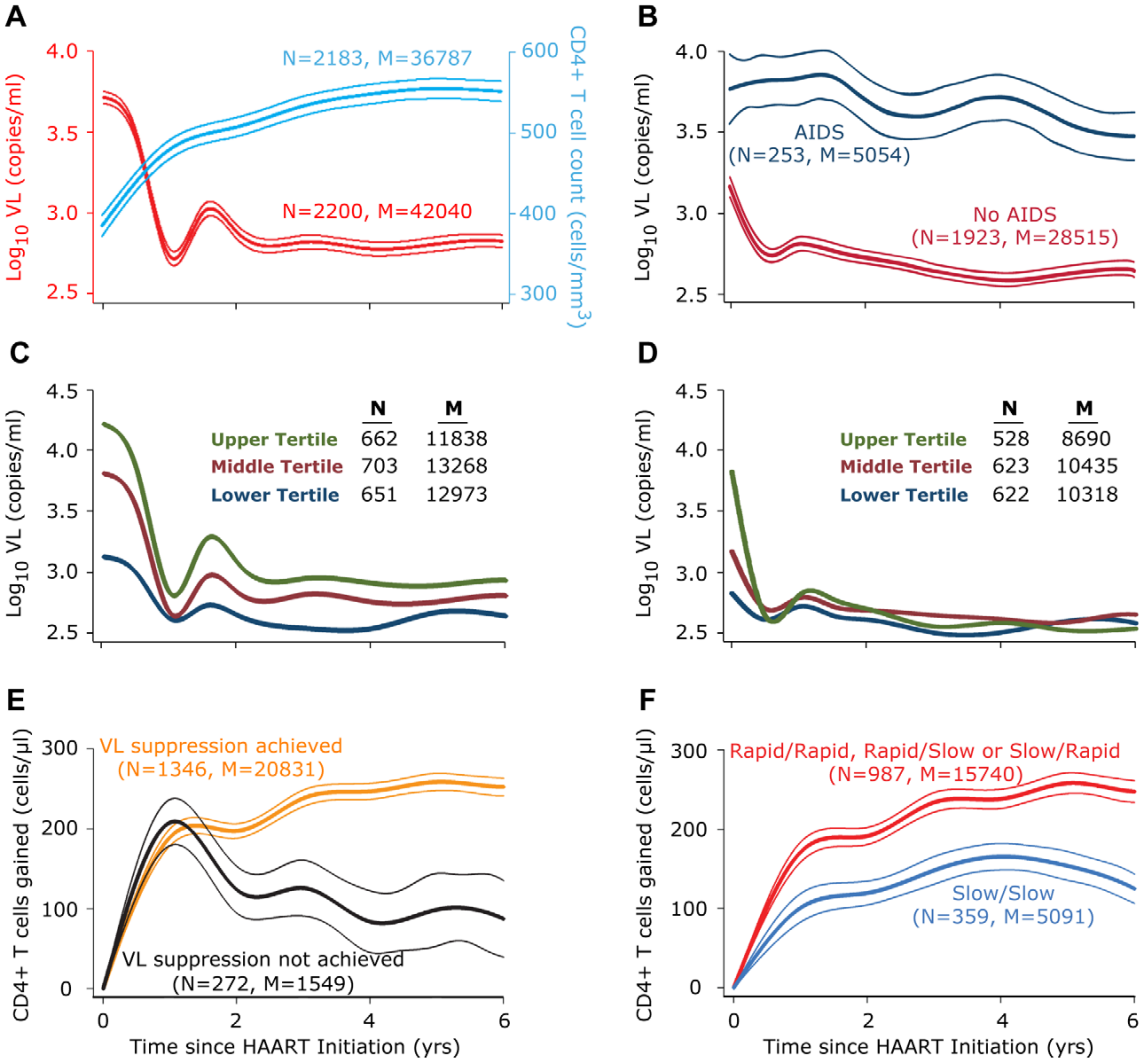
CD4+ T cell count and VL trajectories during HAART. (**A**) Overall (population level) VL and CD4 trajectories after HAART initiation. The curves for CD4 counts (blue and corresponding to the right Y axis) and VL (red and corresponding to the left Y-axis) are superimposed to provide a common temporal view of the trajectories from time of HAART initiation (x-axis). (**B**) VL trajectories in subjects who developed or did not develop AIDS during HAART. (**C–D**) VL trajectories after HAART initiation based on the tertiles of pre-HAART VL in (C) all subjects and (D) those who achieved VL suppression. (**E–F**) CD4+ T cell count trajectories after initiation of HAART according to (E) attainment of VL suppression and (F) VL kinetics among VL suppressers. In panel F, slow/slow indicates subjects who had slow (less than median) rate of VL decay estimated either using all the VL measurements or using those during the first year after HAART initiation. The remaining three groups (rapid/slow, slow/rapid and rapid/rapid) showed similar trajectories and were, therefore, grouped into a single category. All trajectories shown were modeled using non-linear GEE and spline smoothing assuming equal-correlation structure. In panels, A, B, E and F the central thick line represents the mean and the two straddling thin lines represent the edge of the 95% confidence interval band. N, number of subjects; M, number of CD4 or VL measurements.

Characteristics of the 2278 participants who initiated HAART are in Table 1. The average follow up time after HAART for participants was 5.63 years (SD 3.98). Cohort-level non-linear GEE modeling of VL from time-of-HAART initiation in all subjects revealed the following pattern: a precipitous decline in VL during the first year, a temporary rebound at ∼1.6 years post-HAART, followed by a relatively steady-state VL thereafter (Fig. 1A). The VL trajectory of subjects who developed AIDS during HAART versus those who remained AIDS-free differed significantly as a decline in VL after HAART initiation was not observed in patients who developed AIDS (Fig. 1B). In all subjects (Fig. 1C) and in those who attained VL suppression (Fig. 1D), VL trajectories differed according to the tertiles of the pre-HAART VL such that those who started with higher VLs (upper and middle tertiles of pre-HAART VL) displayed a sharper decline in VL than those subjects categorized to the lower pre-HAART VL tertile (Fig. 1C-D, Table S2).

**Table 1:** Characteristics of subjects on HAART studied.

Characteristic	n^a^	Median (IQR) or Percentage
Age at HAART (y)	2275	34.27 (29.15–39.61)
Female gender	2278	188 (8.3%)
Ethnicity	2278	
European Americans		1006 (44.2%)
African Americans		1003 (44.0%)
Hispanic Americans		186 (8.2%)
Others		83 (3.6%)
Baseline CD4 (cells/µl)	2284	466 (330–637)
Nadir CD4 (cells/µl)	2200	278 (167–378)
Time from nadir CD4 to HAART initiation (y)	2200	0.26 (0.03–1.34)
Baseline VL (log_10_ copies/ml)	1951	4.38 (3.75–4.88)
Pre HAART VL (log_10_ copies/ml)	1799	4.35 (3.76–4.85)
Overall VL decay constant (x10^−2^)	2055	2.57 (−1.19–7.06)
VL decay constant during year one of HAART (x10^−2^)	1684	5.22 (−7.8–55.2)
VL slope (log_10_ copies/ml/month) during year one of HAART	1684	0.16 (−0.26–1.42)
Cumulative VL (log_10_ copies*months/ml)	1949	16.31 (7.14–24.94)
Average time to HAART initiation (y)	2278	3.60 (0.46–7.88)
Late HAART era	2278	1579 (69.3%)
Prior use of ARV	2278	1087 (47.7%)
AIDS before HAART initiation	2278	139 (6.1%)
Duration of follow-up on HAART (y)^b^	2278	5.63
VL measurements per individual per year^b^	2278	3.07
CD4 measurements per individual per year^b^	2278	2.65
AIDS after HAART (%)	2278	12.27%
VL suppression		
Ever	2278	1925 (84.5%)
First twelve months	1722	1113 (64.6%)
First six months	1790	1178 (65.8%)
First three months	1294	837 (64.7%)
CD4 slope in first 2 years after HAART (cells/µl/year)	1931	56.6 (−18.0–128.8)
CD4 slope after 2 years of HAART (cells/µl/year)	1532	3.55 (−25.3–34.2)
Mean CD4 count 2 years after HAART (cells/µl)	1560	533.4 (351.1–712)

a n, number of subjects on whom indicated data was available.

b values represent the mean.

The cohort-level trajectories in CD4 counts during HAART revealed two phases of CD4 count changes In phase I, for all subjects initiating HAART there was a rapid increase in CD4 counts during the first two years, followed in phase II by a slower, sustained gain in CD4 cells (Fig. 1A). We stratified the cohort-level changes in CD4 count gains according to whether subjects attained VL suppression (Fig. 1E). This analysis revealed that during the first year of HAART, rapid and similar gains in CD4 counts (∼200 cells on average) were observed in those who did (brown curve) or did not (black curve) attain VL suppression (Fig. 1E). However, in contrast to those who attained VL suppression, the initial gains in CD4 counts were not durable among those who did not achieve VL suppression (Fig. 1E).

### VL kinetic parameters and AIDS risk after HAART

The association of the three VL kinetic parameters and cumulative VL with risk of developing AIDS during HAART was evaluated in separate multivariate models adjusted for length of follow up. For these and the other analyses described later, we dichotomized subjects based on the VL parameters using the median value of the parameter as the cut-off. We included into each multivariate model additional covariates that have been shown to be predictive of immunologic recovery during HAART, including time to VL suppression (Table 2).[26], [33], [34], [35], Nadir CD4 was used as a surrogate for pre-HAART CD4 counts because the median time from the nadir CD4 to HAART initiation was short (Table 1).

**Table 2:** Association of VL parameters with risk of AIDS development after initiation of HAART^a^.

VL parameter	Unadjusted			Adjusted		
	RR	95% CI	p	RR	95% CI	p
VL parameters using all available measurements						
Overall decay constant	1.32	0.96–1.82	0.087	1.38	0.99–1.94	0.058
Decay constant in first year	1.03	0.72–1.47	0.876	1.07	0.74–1.55	0.730
Slope in first year	0.99	0.69–1.42	0.964	1.04	0.72–1.51	0.828
Cumulative VL	2.22	1.56–3.13	<0.001	2.38	1.56–3.62	<0.001
VL parameters by excluding measurements after first AIDS event						
Overall decay constant	1.29	0.94–1.77	0.122	1.35	0.97–1.89	0.080
Decay constant in first year	1.05	0.73–1.50	0.795	1.07	0.73–1.55	0.732
Slope in first year	1.05	0.73–1.49	0.805	1.05	0.72–1.52	0.801
Cumulative VL	2.22	1.57–3.13	<0.001	2.38	1.56–3.62	<0.001

a Results are from a Poisson regression model adjusted for the length of follow-up. Unadjusted results are from the bivariate models with the indicated VL parameter as the predictor and AIDS development as the outcome. All VL parameters were dichotomized based on their respective medians. The RRs are for the association of slow decay (as indicated by less than median decay constants and slope) and high (greater than median) cumulative viral load with AIDS development. Adjusted models are multivariate models that included the following covariates: baseline and nadir CD4 count, pre-HAART VL, time to HAART, age at HAART initiation (per 10 years) and time to VL suppression were included as continuous variables.

The slope and exponential decay constant for VL during the first year of HAART were not predictive of AIDS, whereas a slower overall VL decay showed a statistical trend towards predicting AIDS during HAART, independent of the other covariates (RR 1.38, p = 0.058). A higher than average cumulative VL during HAART (RR = 2.38, 95% CI = 1.56–3.62) was associated with the greatest risk of developing AIDS. These results were similar when the VL parameters were estimated excluding the VL measurements recorded after the AIDS event occurred (Table 2) or when the analyses were restricted to seroconverters only (Table S3).

In separate analyses, where attainment of VL suppression at any point during HAART was replaced with VL suppression by 6 or 12 months, overall VL decay constant was a significant independent predictor of AIDS in patients who attained VL suppression at 6 (RR = 1.65, p = 0.007, 95% CI = 1.14–2.38) and 12 (RR = 1.43, p = 0.055, 95% CI = 0.99–2.05) months, whereas in these models, the VL slope or exponential VL decay during the first year were not predictive of AIDS. The cumulative VL remained highly predictive of AIDS risk in those who attained VL suppression during 6 (RR = 1.96, p = 0.004, 95% CI = 1.24–3.13) and 12 (RR = 2.33, p = 0.001, 95% CI = 1.44–3.80) months of HAART. Collectively, these data indicated that a slow overall VL exponential decay and a high cumulative VL during HAART increased AIDS risk after initiation of HAART.

### VL parameters and CD4 Recovery

We next determined whether the VL kinetic and other parameters that were included in the models to assess AIDS risk during HAART also associated with the rate of CD4 gain (Table 3). We found that the VL parameters predicted different aspects of CD4 count recovery even after accounting for factors that we found to be highly predictive of AIDS risk, including prior history of AIDS, nadir CD4, age at HAART initiation and time to VL suppression. The overall decay rate constant was not predictive of rate of CD4 gain in the first 2 years, but was significantly associated with the rate of CD4 gain after two years of HAART, the mean CD4 count two years after HAART, and the overall gain of CD4 cells (Table 3). The decay constant and VL slope in the first year of HAART were mostly predictive of the rate of CD4 cell gain during the first two years and the overall gain in CD4 cells (Table 3). By contrast, the cumulative VL was only predictive of rate of CD4 gains after 2 years and not the overall gain in the CD4 count (Table 3).

**Table 3:** Association of VL parameters with CD4 recovery after HAART initiation in subjects who did not develop AIDS.

Outcomes and Adjustment	Overall decay constant		Decay constantin first year		Slope in first year		Cumulative VL	
	Coeff (SE)	*p*	Coeff (SE)	*p*	Coeff (SE)	*p*	Coeff (SE)	*p*
**Model 1: Rate of CD4 gain in first 2 years (cells/µl/year)**								
All subjects	6.98 (9.29)	0.453	32.01 (10.43)	0.002	32.69 (10.43)	0.002	−2.52 (11.38)	0.824
VL suppressors	3.32 (9.29)	0.721	33.22 (10.42)	0.001	33.91 (10.42)	0.001	−0.06 (11.60)	0.996
Seroconverters	1.12 (10.54)	0.915	33.09 (11.67)	0.005	33.12 (11.67)	0.005	9.39 (13.00)	0.470
**Model 2: Rate of CD4 gain after 2 years (cells/µl/year)**								
All subjects	40.59 (8.46)	<0.001	−5.29 (9.86)	0.592	−6.34 (9.87)	0.521	52.19 (10.87)	<0.001
VL suppressors	41.31 (8.61)	<0.001	−6.43 (10.02)	0.521	−7.50 (10.03)	0.455	52.92 (11.09)	<0.001
Seroconverters	43.59 (9.87)	<0.001	−1.81 (11.34)	0.873	−3.19 (11.36)	0.779	50.59 (12.47)	<0.001
**Model 3: Mean CD4 count 2 years after HAART (cells/µl)**								
All subjects	40.21 (14.40)	0.004	33.67 (16.00)	0.036	24.42 (16.03)	0.128	−4.54 (17.52)	0.795
VL suppressors	24.97 (14.10)	0.077	23.87 (16.02)	0.137	14.39 (16.05)	0.370	10.31 (17.65)	0.559
Seroconverters	32.08 (15.66)	0.041	34.36 (17.58)	0.051	22.93 (17.62)	0.194	9.72 (19.23)	0.613
**Model 4: Overall gain of CD4 cells (cells/µl)**								
All subjects	53.62 (17.45)	0.002	41.77 (18.80)	0.027	38.77 (18.81)	0.040	1.78 (24.11)	0.941
VL suppressors	51.06 (17.49)	0.004	40.35 (18.96)	0.034	37.53 (18.95)	0.048	13.13 (24.55)	0.593
Seroconverters	43.78 (19.40)	0.025	47.42 (20.54)	0.021	42.31 (20.57)	0.040	29.31 (26.49)	0.269

The results are from multivariate linear regression models, and shown are the linear regression coefficients and their standard errors along with significance values. Each model set has three models for the indicated subjects. Each model is adjusted for covariates that in previous analyses were shown to associate with risk of AIDS and were age at HAART initiation, gender, time from entry into cohort to HAART initiation, African American ethnicity, previous receipt of ARV, AIDS prior to HAART, pre-HAART VL, time to VL suppression and late HAART era (after 2000; Marconi et al).

The aforementioned data suggested that a slower VL decay during the first year of HAART is associated with both a reduced rate of CD4 gain in the first two years of HAART and overall gain in CD4 cells (Table 3). By contrast, a slower overall VL decay is more predictive of a reduced rate of CD4 gain after 2 years of HAART, lower mean gains in CD4 counts after 2 years of HAART as well as a reduced overall gain in CD4 cells (Table 3). On the basis of these results, we posited that VL suppressers who had a slow VL decay in the first year of HAART and the entire therapy course (overall) would fare the worst with respect to CD4 recovery. To test this, we categorized VL suppressors into two groups: those with a slow decay in the first year and slow overall decay were categorized into one group, whereas the remainder (rapid/rapid, rapid/slow, slow/rapid decay in the first year and overall decay, respectively) were grouped together because they had very similar CD4 count trajectories (data not shown). Subjects categorized to the slow early/slow overall decay group were similar to other subjects with respect to age at HAART initiation, ethnicity and nadir CD4 (all p values >0.2).

Notably, VL suppressers categorized to the slow/slow decay group had a significantly muted CD4 recovery during HAART compared with all other subjects (Fig. 1F). Concordantly, VL suppressors categorized to the slow/slow decay category had a slower rate of CD4 recovery in the first two years (54.6 vs. 80.2 cells/µL/yr, p = 0.02) and after two years (−14.8 vs. 16.5 cells/µL/yr, p = 0.002) of HAART, a lower mean CD4 count after two years (564.6 vs. 614.8 cells/µL, p = 0.005) and a lower absolute CD4 gain (133 vs. 195.4 cells/µL p = 0.001). We also conducted these analyses for subjects who achieved VL suppression within 6 and 12 months and found highly concordant results (data not shown).

## Discussion

Not all patients on HAART display robust CD4 cell gains, despite VL suppression.[11], [12], [13], [14], [15], [16] This has been attributed previously to factors such as pre-HAART VL and nadir CD4, age at HAART initiation, and depth of and time to VL suppression. In this study, we modeled the VL decay and cumulative VL and applied these relatively unique parameters to a large well-characterized cohort in order to determine whether these factors were associated with AIDS risk and CD4 recovery during HAART independent of currently recommended benchmarks of VL suppression at 6 and 12 months.[10] In the participants that we evaluated, the initiation of HAART was associated with a predictable decline in VL that was concomitantly associated with an increase in CD4 counts. Subjects who did or did not achieve VL suppression both experienced, on average, a gain of 200 CD4 cells/µL during the first year of HAART. However, in contrast to those who attained VL suppression, these gains were not sustainable among non-VL suppressers. Notwithstanding the importance of attaining VL suppression or minimizing the time to VL suppression, our data show that in addition to these endpoints, both a slow early (first year of HAART) and slow overall (during entire treatment period) VL decay were independently associated with both a slower rate of and lower absolute CD4 cell gain during HAART. Furthermore, a slower overall VL decay in those who attained VL suppression within 6 and 12 months of HAART initiation, and a higher cumulative VL during HAART were each independent predictors of increased AIDS risk during HAART. These findings suggest that the patterns of VL decay are important factors in addition to VL suppression for CD4 reconstitution and risk of AIDS during HAART.

The pre-HAART VL predicted the subsequent rate of decay during the first year of HAART. Since most patients were able to achieve suppression by 6–12 months, it is not surprising that the rate of decay would be greater for patients with higher initial VLs. This may also suggest that patients with higher initial VLs have a larger proportion of actively replicating, productively-infected cells that are more susceptible to HAART. This is consistent with previous studies that examined decay for patients receiving HAART.[39] It was also intriguing that the cohort-level analyses also revealed that after the initial precipitous decline in VL there was a transient rebound, regardless of initial VL (Fig. 1C). This is due to a combination of individual profiles including a proportion of patients experiencing virologic rebound with subsequent resuppression, a small percentage experiencing rebound and not achieving resuppression, and some patients experiencing blips. It is unclear if this temporary, population-level rebound represents a specific temporal relationship with average time to medication fatigue and/or the development of virologic drug resistance as nearly half of treated patients experience a change in therapy around this time both as reported in this cohort[26] and elsewhere.[40] Even in the absence of complete rebound from poor adherence or drug resistance, periods of increased replication can occur due to pharmacologic changes or altered drug activity in a particular compartment.[29] Modeling data from structured treatment interruption trials have shown that parametric resonance, such as that seen in our study, can occur even in the absence of drug resistance and complete virologic rebound.[41] This phenomenon can be seen when a system undergoing small oscillations over time (such as during the dynamic equilibrium of viral load setpoint) undergoes a significant dampening (HAART initiation) and then experiences brief periods of external perturbation (brief treatment interruptions).

Although the importance of early virologic suppression and virologic failure on CD4 recovery has been well-described,[21], [22], [24], [42], much less is known about the impact of rate of decay or detectable VL after initial suppression of VL on CD4 recovery.[25], [38], [45], [46], [47], [48] In this study, we incorporated several of these elements into a single parameter of overall virologic decay. This parameter provides information on the early trajectory as well as the durability of the VL response after the initial decay. We also evaluated cumulative VL because it could be argued that it is the overall exposure to virus that influences CD4 recovery and AIDS.[38], [49], [50], [51] We found that cumulative VL was a stronger predictor of AIDS risk than CD4 recovery after HAART initiation. Additionally, VL decay or slope within the first year of HAART was not predictive of AIDS, whereas the overall VL decay predicted AIDS even among patients who attained VL suppression during 6 and 12 months of HAART. Thus, it is striking that the risk of AIDS is not impacted by the initial rapid phase of virologic decay, but rather by longitudinal assessments such as the overall decay or cumulative VL. These findings suggest that risk of AIDS during HAART is more sensitive to the VL over time rather than events that occur during the first year of HAART as has been suggested previously.[52], [53], [54], [55] In contrast to this study which examined the impact after HAART, Cole et al. recently found that cumulative VL predicted AIDS or death in absence of HAART independent of known risk factors in the Multicenter AIDS Cohort Study.[56] As the number of serious non-AIDS events during HAART increases relative to the number of AIDS events over time, it will be important to determine the association of overall virologic decay and cumulative VL with serious non-AIDS events as has been demonstrated with cancer[57], [58] and renal impairment.[59] This data would also suggest that perhaps the cumulative VL even prior to HAART could be associated with clinical events during HAART, supporting the notion that earlier diagnosis and treatment would further reduce the number of these adverse outcomes.

Even among subjects who attained VL suppression, and after adjustment for time to VL suppression, a slow overall VL decay was predictive of late/long-term CD4 changes (rate of CD4 gain, mean CD4 count after two years of HAART, and overall gain in CD4 cells), but not early CD4 changes (rate of CD4 gain during the first two years of HAART). In contrast, a slower VL decay within the first year of HAART associated with early but not later/long-term CD4 changes during HAART. These results suggest that, although among VL suppressers the pace and extent of CD4 gain during the early phases of HAART may be highly correlated with both the early and overall VL decay patterns, durable gains in CD4 cells after two years of HAART may be highly dependent on the overall VL decay pattern. These findings demonstrated that VL suppressors could be stratified into two categories such that those with both a slow early and overall VL decay (slow early/slow overall decay) will achieve CD4 recovery, but the gain in CD4 cells would be significantly muted relative to all other subjects (Fig. 1F). Because studies have identified polymorphisms that track the durability of CD4 recovery, it will be important to evaluate whether the patterns of VL decay are in part related to such host factors.[11]

The association of the extent of CD4 recovery was strongest with the overall VL decay and not the first year VL decay or the cumulative VL, suggests that these VL parameters may be capturing different aspects of VL changes during HAART (early trajectory and maintenance of suppression). The overall decay provides information throughout the duration of treatment and is not limited to one year of information. Hence, it is probable that the decay pattern occurring after virologic suppression (third phase of VL decay)[60] indexed to the decay patterns that occur immediately after HAART initiation[23] together contribute to the ability of a patient to experience durable immune reconstitution. It remains unclear if the latter phases of immune reconstitution are affected by “blips” or primarily by more substantial viral rebound.[61], [62]

In contrast to the overall decay pattern, the cumulative VL is a coarse measure of overall VL burden (total virus exposure) during HAART and does not account for VL decay patterns. For example, a patient who suppresses early but has late rebound might have a comparable cumulative VL to that of a patient with predominantly late virologic suppression. This may partly explain why this parameter as computed may not associate strongly with CD4 recovery. However, another explanation hinges on the use of detectable VLs to compute this parameter. Certainly, patients with complete or repetitive virologic rebounds may experience a loss of CD4 recovery; however, the vast majority of patients in this cohort achieved suppression within the first year and the rate of rebound was low.[26] Therefore, at the frequency of available measurements, the cumulative VL may not capture some of the intermittent or ongoing low-level viremia during HAART which may represent actual viral replication in the setting of periodic HAART interruption. Hence, it is conceivable that computation of the cumulative VL using more frequent measurements and/or single copy assays that assess VL below the detectable threshold of commercial assays might reveal that the cumulative VL is a more sensitive marker of not only AIDS risk but also CD4 recovery.

We investigated a large number of prospectively evaluated subjects who have equal access to healthcare and high rates of adherence to HAART.[26], [30] This afforded an excellent opportunity to observe the impact of virologic parameters on CD4 recovery in a setting outside of a clinical trial, making these results more generalizable to the HIV-infected population at large. There are some limitations of this study. This study did not attempt to dissect the components of the VL decay[24], [63] and determine what baseline and subsequent factors contribute to these components. For example, different regimens, the existence of drug resistance, variable pharmacokinetics and adherence patterns can result in different rates for the first and second phases of VL decay, respectively.[24], [28] To this end, we used HAART era as a covariate in the multivariate model to adjust for regimen potency and prior single or dual ART. Furthermore, although rates of adherence in this cohort[26] are high, the rationale of these analyses was not to understand the impact of adherence on the rate of decay but instead how the decay patterns alone influence subsequent clinical/immunologic outcomes regardless of the level of adherence which in a clinical setting can often be unreliable. We also acknowledge that we studied a total of 52 multivariate models (shown in Tables 2 and 3) and at a global type I error rate of 0.05, 2–3 observed associations are likely to be erroneous. Given the fact, however, that we observed a total of 23 associations to be significant at 0.05 type I error rate, our study results are unlikely to have been influenced by false positive associations due to multiple testing. Finally, although the impact of drug resistance and pharmacokinetic interactions was not examined in this study, prior ARV use was used as a surrogate marker of baseline resistance in the multivariate models.

In summary, our findings underscore that the early and overall patterns of VL decay among VL suppressed patients is an independent determinant of CD4 recovery. In addition, the cumulative VL is a determinant of AIDS risk during HAART. Thus, inter-individual differences in VL decay patterns may partly explain the wide variability in CD4 recovery even among those individuals achieving VL suppression within the recommended timeframe. These results also suggest that regimens that produce the most rapid virologic decay and durable suppression could lead to better clinical/immunologic responses. These parameters could be further developed to enhance clinical trial assessment of ARV regimens and assist clinicians with identifying patients at risk for adverse events beyond standard indicators.

## Supporting Information

Table S1 **Definitions for various parameters used in this study.**
(DOCX)

Table S2 **Modeling of VL kinetics based on tertiles of pre-HAART VL.**
(DOCX)

Table S3 **Association of VL parameters with risk of AIDS development after initiation of HAART among seroconverters.**
(DOCX)

Note S1 **Statistical concepts in VL parameter estimation.**
(DOCX)
