# Supplementary material for: Cumulative Viral Load and Virologic Decay Patterns after Antiretroviral Therapy in HIV-Infected Subjects Influence CD4 Recovery and AIDS
Source: PLoS One. 2011 May 20;6(5):e17956. doi: 10.1371/journal.pone.0017956 (PMC3098832; doi:10.1371/journal.pone.0017956)
Supplement: Table S1 — Definitions for various parameters used in this study. (DOCX) [file pone.0017956.s001.docx]

**Table S1. Definitions for various parameters used in this study.**

| **Parameter** | **Definition** |
| --- | --- |
| Seroconverter | A subject having a documented HIV seronegative date prior to the first positive HIV date. |
| Date of seroconversion | Mid-point of the interval between a documented HIV seronegative date and the first positive HIV date. |
| Anti-retroviral therapy (ARV) | Therapy, typically with one or two drugs, not meeting the definition of HAART. |
| Highly active anti-retroviral therapy (HAART) | 1. Two or more nucleoside reverse transcriptase inhibitors (NRTIs) in combination with at least one protease inhibitor (PI) or one non-nucleoside reverse transcriptase inhibitor (NNRTI) or 2. One NRTI with at least one PI and one NNRTI or 3. An abacavir or tenofovir containing regimen of three or more NRTIs in the absence of both PIs and NNRTIs. |
| Time to HAART | Interval between documented date of HIV positivity and HAART start date. |
| Late HAART era | Calendar period beginning 1 Jan 2000 after which HAART was initiated. |
| Baseline viral load (VL) | First VL measured after the first documented date of seroconversion or enrollment. |
| Baseline CD4 T cell count | First CD4 T cell count measured after the first documented date of seroconversion or enrollment. |
| Nadir CD4 count | The lowest CD4 T cell count observed over the entire duration of follow-up prior to initiation of HAART. |
| Pre-HAART VL | Mean VL during 1 year preceding HAART initiation. |
| Cumulative VL | The area under the VL-time curve using trapezoidal rule after HAART initiation including the baseline VL. |
| VL suppressors | Subjects who had at least one VL measurement <400 copies/mL over the duration of the follow-up on HAART. |
| Time to VL suppression | Time from the date of initiation of HAART to the first VL measurement below <400 copies/mL. |
| Overall VL decay constant | Estimated parameter λ from a regression analysis of VL measurements after HAART initiation over entire HAART duration by fitting the exponential function of the form VL = *e^-λt^*. See Note S1 for details. |
| VL decay constant during first year of HAART | Estimated parameter λ from a regression analysis of VL measurements after HAART initiation over time during the first year by fitting the exponential function of the form VL = *e^-λt^*. This analysis was restricted to those subjects who had at least 3 VL measurements during the first year after HAART initiation. See Note S1 for details. |
| VL slope during first year of HAART | Estimated parameter m from a regression analysis of VL measurements after HAART initiation over time by fitting a linear function of the form VL = *mt + c.*  This analysis was restricted to those subjects who had at least 2 VL measurements during the first year after HAART initiation. See Note S1 for details. |
| Mean phase II of CD4 count recovery | Average of all CD4 T cell count measurements two years after HAART start date. |
| Overall CD4 gain | Post-HAART CD4 count – nadir CD4 count. |
| Slope of CD4 gain in phase I of CD4 count recovery (first 2 years of HAART) | Estimated parameter m from a regression analysis of CD4 T cell count measurements after HAART initiation over time by fitting a linear function of the form CD4 count = *mt + c.*  This analysis was restricted to those subjects who had at least 2 CD4 T cell count measurements during the first two years after HAART initiation. |
| Slope of CD4 gain during phase II of CD4 count recovery | Estimated parameter m from a regression analysis of CD4 T cell count measurements after HAART initiation over time by fitting a linear function of the form CD4 count = *mt + c.*  This analysis was restricted to those subjects who had at least 2 CD4 T cell count measurements after two years after HAART initiation. |
