# Supplementary material for: Cumulative Viral Load and Virologic Decay Patterns after Antiretroviral Therapy in HIV-Infected Subjects Influence CD4 Recovery and AIDS
Source: PLoS One. 2011 May 20;6(5):e17956. doi: 10.1371/journal.pone.0017956 (PMC3098832; doi:10.1371/journal.pone.0017956)
Supplement: Table S2 — Modeling of VL kinetics based on tertiles of pre-HAART VL. (DOCX) [file pone.0017956.s002.docx]

**Table S2:** Modeling of VL kinetics based on tertiles of pre-HAART VL.

| **Model** |  | **Tertile** |  | **n** |  | **M** |  | **Coeff** |  | **95% CI** |  | ***P*** |
| --- | --- | --- | --- | --- | --- | --- | --- | --- | --- | --- | --- | --- |
|  |  |  |  |  |  |  |  |  |  |  |  |  |
| Linear GEE |  | Lower |  | 651 |  | 12973 |  | -0.033 |  | -0.037 – -0.028 |  | Reference |
|  |  | Middle |  | 703 |  | 13268 |  | -0.067 |  | -0.072 – -0.062 |  | 9.6x10^-21^ |
|  |  | Upper |  | 662 |  | 11838 |  | -0.077 |  | -0.083 – -0.071 |  | 8.2x10^-28^ |
| Linear GEE during the first year |  | Lower |  | 618 |  | 2079 |  | -0.087 |  | -0.180 – -0.006 |  | Reference |
|  |  | Middle |  | 661 |  | 2353 |  | -0.352 |  | -0.464 – -0.239 |  | 0.0004 |
|  |  | Upper |  | 636 |  | 2438 |  | -0.886 |  | -1.010 – -0.763 |  | 3.5x10^-23^ |
| Exponential GEE |  | Lower |  | 651 |  | 12973 |  | -0.018 |  | -0.019 – -0.016 |  | Reference |
|  |  | Middle |  | 703 |  | 13268 |  | -0.028 |  | -0.030 – -0.026 |  | 1.6x10^-15^ |
|  |  | Upper |  | 662 |  | 11838 |  | -0.031 |  | -0.033 – -0.029 |  | 5.9x10^-21^ |
| Exponential GEE in the 1st year |  | Lower |  | 618 |  | 2079 |  | -0.046 |  | -0.079 – -0.013 |  | Reference |
|  |  | Middle |  | 661 |  | 2353 |  | -0.148 |  | -0.185 – -0.111 |  | 5.2x10^-5^ |
|  |  | Upper |  | 636 |  | 2438 |  | -0.322 |  | -0.360 – -0.284 |  | 4.5x10^-26^ |

Subjects were categorized according to the tertiles of pre-HAART VL, and the lower tertile of VL served as the reference category.
