# Supplementary material for: Cumulative Viral Load and Virologic Decay Patterns after Antiretroviral Therapy in HIV-Infected Subjects Influence CD4 Recovery and AIDS
Source: PLoS One. 2011 May 20;6(5):e17956. doi: 10.1371/journal.pone.0017956 (PMC3098832; doi:10.1371/journal.pone.0017956)
Supplement: Table S3 — Association of VL parameters with risk of AIDS development after initiation of HAART among seroconverters. (DOCX) [file pone.0017956.s003.docx]

**Table S3: Association of VL parameters with risk of AIDS development after initiation of HAART among seroconverters**

| **Covariate** |  | **Overall**  **Decay Constant**  **(n = 1,184)** |  | **Decay constant**  **during first year**  **(n = 1029)** |  | **Slope**  **during first year**  **(n = 990)** |  | **Cumulative VL**  **(n = 1,139)** |
| --- | --- | --- | --- | --- | --- | --- | --- | --- |
|  |  |  |  |  |  |  |  |  |
| VL kinetic parameter |  | 1.72 (1.11 – 2.65), 0.014 |  | 1.07 (0.68 - 1.67), 0.768 |  | 1.02 (0.65 - 1.58), 0.940 |  | 2.49 (1.42 - 4.38), 0.001 |
| Log_10_ (pre-HAART VL) |  | 1.36 (0.99 – 1.86), 0.055 |  | 1.28 (0.89 - 1.83), 0.176 |  | 1.28 (0.89 - 1.83), 0.180 |  | 1.25 (0.91 - 1.71), 0.174 |
| Baseline CD4 T cell count |  | 0.99 (0.90 - 1.10), 0.914 |  | 1.02 (0.92 - 1.13), 0.735 |  | 1.02 (0.92 - 1.12), 0.750 |  | 1.00 (0.91 - 1.11), 0.950 |
| Nadir CD4 count |  | 0.98 (0.99 - 0.99), 0.028 |  | 1.00 (1.00 - 1.00), 0.107 |  | 1.00 (1.00 - 1.00), 0.108 |  | 1.00 (1.00 -1.00), 0.017 |
| Female gender |  | 0.33 (0.08 – 1.36), 0.125 |  | 0.45 (0.11 - 1.84), 0.265 |  | 0.45 (0.11 - 1.85), 0.268 |  | 0.36 (0.09 - 1.46), 0.151 |
| African ancestry |  | 1.52 (1.01 – 2.29), 0.046 |  | 1.49 (0.95 - 2.31), 0.082 |  | 1.49 (0.95 - 2.32), 0.081 |  | 1.49 (0.98 - 2.24), 0.060 |
| Age at HAART initiation |  | 0.99 (0.95 - 1.01), 0.343 |  | 0.98 (0.95 - 1.02), 0.334 |  | 0.98 (0.95 - 1.02), 0.326 |  | 0.99 (0.96 - 1.02), 0.385 |
| Prior use of ARV |  | 0.79 (0.44 - 1.43), 0.443 |  | 0.85 (0.46 - 1.59), 0.611 |  | 0.85 (0.95 - 1.02), 0.609 |  | 0.78 (0.43 - 1.43), 0.426 |
| Prior AIDS |  | 2.14 (1.22 - 3.74), 0.008 |  | 2.78 (1.55 - 4.97), 0.001 |  | 2.77 (1.55 - 4.96), 0.001 |  | 1.93 (1.10 - 3.38), 0.021 |
| VL suppressor |  | 0.30 (0.15 - 0.58), <0.001 |  | 0.17 (0.08 - 0.35), <0.001 |  | 0.17 (0.08 - 0.35), <0.001 |  | 0.34 (0.18 - 0.65), 0.001 |
| Late HAART era |  | 0.68 (0.38 - 1.21), 0.191 |  | 0.74 (0.39 - 1.41), 0.362 |  | 0.74 (0.39 - 1.42), 0.369 |  | 0.87 (0.45 - 1.69), 0.691 |
| Time to HAART |  | 1.09 (1.01 - 1.17), 0.034 |  | 1.09 (1.00 - 1.18), 0.046 |  | 1.09 (1.00 - 1.18), 0.042 |  | 1.08 (1.00 - 1.16), 0.042 |
| Time to VL suppression |  | 1.13 (1.05 - 1.22), 0.001 |  | 1.14 (1.05 - 1.23), 0.002 |  | 1.14 (1.05 - 1.23), 0.002 |  | 1.14 (1.06 - 1.23), <0.001 |
