# Supplementary material for: Cumulative Viral Load and Virologic Decay Patterns after Antiretroviral Therapy in HIV-Infected Subjects Influence CD4 Recovery and AIDS
Source: PLoS One. 2011 May 20;6(5):e17956. doi: 10.1371/journal.pone.0017956 (PMC3098832; doi:10.1371/journal.pone.0017956)
Supplement: Note S1 — Statistical concepts in VL parameter estimation. (DOCX) [file pone.0017956.s004.docx]

**Note S1: Statistical concepts in VL parameter estimation**

We aimed to fit linear and exponential models to the longitudinal VL data post-HAART. We fitted three regression models: the first one fitted an exponential model of the form log VL = *e^-λt^*, where λ is the decay constant and t is time since HAART initiation; the second was the same model fitted to VL measurements within the first year only and the third one was a linear regression model of the form VL = *mt + c*, where m is the slope of VL decrease, t is the time since HAART initiation and c is the intercept. The third model was also restricted to the VL measurements during the first year only.

There were clinical and statistical reasons overriding the choice of these three models. First, we argued that the VL decay is a dynamic and continuous process and that the contiguous phases referred to in the literature actually represent a single process that results in two seemingly different rates of decay. For this reason we chose to fit an exponential decay model that would fit the whole dataset. Second, from a clinical perspective, the clinician can draw maximum benefit if VL trajectory during the first year of treatment can be modeled. We therefore decided to restrict the exponential model to the observations made during first year of HAART only and tried to associate that with development of AIDS. However, this short period of one year poses two statistical challenges: first, there may be fewer number of measurements that can reduce the power and fit of the exponential decay model; and second during this time, the decay can be expected to be so rapid as to masquerade as a straight line. For this reason, we also decided to examine if a simple linear relationship between VL and time can explain the variability in outcomes.

The method of estimation of the parameters is shown here using example
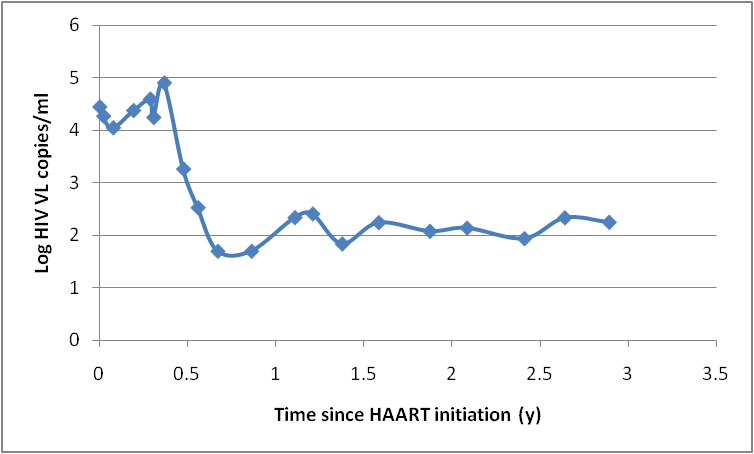
 from an actual patient in the dataset. In this patient there were 20 VL measurements over 3 years of HAART. Highest VL was seen 4.4 months after HAART initiation, then the VL declined rapidly and the patient achieved VL suppression (<400 copies/ml) at 6.7 months and the VL went below the limit of detectability (50 copies/ml) in 8.1 months. When we fitted the overall decay model to all these 20 observations, we found that the decay constant was 0.28 (95% CI 0.13 - 0.42), p = 0.001 and the model fit r^2^ was 44%. When we restricted the model to the 11 VL measurements during the first year, the decay constant was 1.21 (95% CI 0.65 - 1.77), p = 0.001 and the model fit r^2^ rose to 70%. The better model fit is expected since the VL measurements were in a steady state after the first year. When we fitted the linear model to the 11 VL measurements during the first year the slope was estimated to be -3.51 (95% CI -3.26 - -1.76), p=0.001 and the model was still high (66%).

Although, we fitted the exponential model to all the VL measurements on all subjects, it is at least possible theoretically that each patient may not exhibit VL dynamics that fit the VL data well. Shown below is an example from another patient who achieved VL suppression long after HAART was initiated.
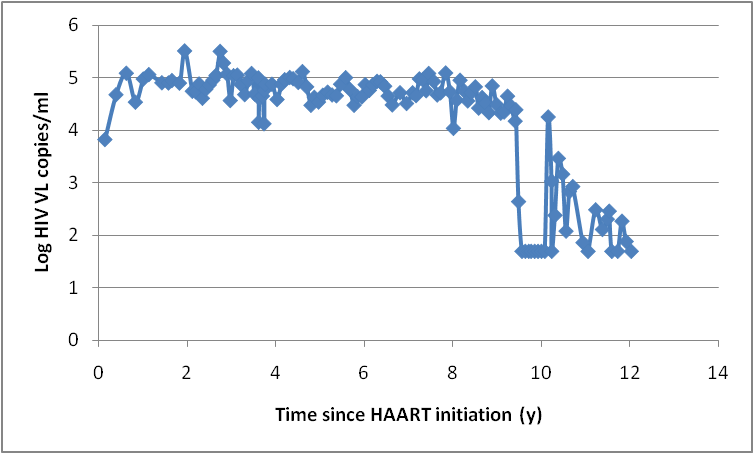
In this patient our estimates were as follows: overall decay constant 0.08 (95% CI 0.06 - 0.09), p<0.001, r^2^ = 47%; decay constant within the first year -0.24 (95% CI -0.65 - 0.18), p = 0.165, r^2^ = 37%; and slope during first year 1.03 (95% CI -0.81 - 2.88), p = 0.172, r^2^ = 35%. Together, these three parameters describe the trajectory of VL in this patient such that while there was an overall exponential decay, it was very slow (compare the decay constant of 0.28 for the previous patient with 0.08 for this patient) and that during the first year neither exponential nor linear models could fit the observed data. Indeed, as can be seen in the trajectory the VL actually increased during the first year of HAART.

A potential confounder to the estimates of the parameters was the development of an AIDS event. We therefore re-estimated all the VL parameters by including only those VL measurements that were obtained prior to the diagnosis of AIDS.
